# Supplementary material for: A chiral fermionic valve driven by quantum geometry
Source: Nature. 2025 Dec 31;649(8095):47–52. doi: 10.1038/s41586-025-09864-5 (PMC12756060; doi:10.1038/s41586-025-09864-5)
Supplement: Supplementary file 1 — The file has two sections on semiclassical Boltzmann formalism and symmetry analysis for competing mechanisms. The first section provides the nonlinear Hall responses up to third order in a Boltzmann transport formalism. The second section provides a symmetry analysis to determine the allowed responses based on the crystalline symmetries [file 41586_2025_9864_MOESM1_ESM.pdf]

nature portfolio

https://doi.org/10.1038/s41586-025-09864-5

Supplementary information

A chiral fermionic valve driven by quantum geometry

In the format provided by the authors and unedited

Supplementary Information for
Chiral Fermionic Valve driven by Quantum Geometry

Anvesh Dixit[1], Pranava K. Sivakumar[1], Kaustuv Manna[2,3], Claudia Felser[2], and Stuart S. P. Parkin[1]

[1]Max Planck Institute of Microstructure Physics, Halle (Saale), Germany
[2]Max Planck Institute for Chemical Physics of Solids, Dresden, Germany
[3]Department of Physics, Indian Institute of Technology Delhi, New Delhi, India

Correspondence: claudia.felser@cpfs.mpg.de, stuart.parkin@mpi-halle.mpg.de

1 Semiclassical Boltzmann formalism for NLH effect

In this section we provide a preliminary analysis of non-linear Hall effects up to third order in a semi-classical Boltzmann formalism, following the line of reasoning in [1, 2]. The Boltzmann equation for a species of electrons (i.e. within one band) in an external electric field $\mathbf{E}(t)$ is given by:

$$\frac{d\mathbf{r}}{dt} \cdot \nabla_{\mathbf{r}} f + \frac{d\mathbf{k}}{dt} \cdot \nabla_{\mathbf{k}} f + \frac{\partial f}{\partial t} = \left(\frac{\partial f}{\partial t} \right)_{\text{coll}}. \tag{S1}$$

In the relaxation-time approximation, we can take $\left(\frac{\partial f}{\partial t}\right)_{\text{coll}} \approx \frac{f_0 - f}{\tau}$ where f_0 is the equilibrium distribution function. This approximation is valid if $\hbar/\Delta \ll \tau \ll L_{\text{sample}}/v_F$, where L_{sample} is the length of the sample, v_F the Fermi velocity and Δ is the energy gap to neighbouring bands. So there must be enough scattering to prevent coherent effects, but not too much to destroy the single-band picture. We can then substitute the semi-classical equations of motion (in the absence of a magnetic field) for electrons in one band, with the anomalous velocity correction due to the Berry curvature $\mathbf{\Omega}$ of the band:

$$\frac{d\mathbf{r}}{dt} = \frac{1}{\hbar} \nabla_{\mathbf{k}} \varepsilon_{\mathbf{k}} + \frac{e}{\hbar} \mathbf{E} \times \mathbf{\Omega},$$
$$\frac{d\mathbf{k}}{dt} = -\frac{e}{\hbar} \mathbf{E}.$$

These semiclassical equations of motion describe a system of electron wave-packets formed from superposing various Bloch states, and is therefore valid when the localization length of these wave-packets is much larger than the lattice spacing, but still smaller than the electric field wavelength. We also assume $\hbar\omega \ll \Delta$ where ω the frequency of the external electric field. Assuming a homogeneous system so that $\nabla_{\mathbf{r}} f = 0$, we find that:

$$\frac{\partial f(\mathbf{k}, t)}{\partial t} - \frac{e}{\hbar} \mathbf{E}(t) \cdot \nabla f(\mathbf{k}, t) = \frac{f_0(\mathbf{k}) - f(\mathbf{k}, t)}{\tau}.$$

From now on we will write $\frac{\partial}{\partial t} \equiv \partial_t$ and $\frac{\partial}{\partial k_\alpha} \equiv \partial_\alpha$. Let us decompose f into a power series in E so $f - f_0 = f_1 + f_2 + f_3 + ...$, so that f_i is proportional to ith power of E. Then we obtain a recursive system of differential equations:

$$(1 + \tau\partial_t) f_n - \frac{e\tau}{\hbar} E_\alpha \partial_\alpha f_{n-1} = 0, \tag{S2}$$

with f_0 being the equilibrium Fermi-Dirac distribution. We take an AC field $\mathbf{E} = \mathrm{Re}\{\mathcal{E} e^{i\omega t}\}$, and therefore we modify the distribution function as $f_n = \mathrm{Re}\{\tilde{f}_n\}$.

The first order correction is given as:

$$\tilde{f}_1 = \tilde{f}_1^\omega e^{i\omega t},\ \tilde{f}_1^\omega = \frac{e\tau/\hbar}{1 + i\omega\tau} \mathcal{E}_\alpha \partial_\alpha f_0 \tag{S3}$$

which gives rise to the familiar Drude conductivity formula for the first harmonic current generation. The second order corrections contain zeroth and second harmonics:

$$\tilde{f}_2 = \tilde{f}_2^0 + \tilde{f}_1^{2\omega} e^{2i\omega t},\ \tilde{f}_2^0 = \frac{(e\tau/\hbar)^2}{2(1 + i\omega\tau)} \mathcal{E}_\alpha^* \mathcal{E}_\beta \partial_\alpha \partial_\beta f_0, \tag{S4}$$
$$\tilde{f}_2^{2\omega} = \frac{(e\tau/\hbar)^2}{2(1 + i\omega\tau)(1 + 2i\omega\tau)} \mathcal{E}_\alpha \mathcal{E}_\beta \partial_\alpha \partial_\beta f_0$$

Finally the third order correction is $f_3 = \mathrm{Re}\{\tilde{f}_3^\omega e^{i\omega t} + \tilde{f}_3^{3\omega} e^{3i\omega t}\}$ where

$$\tilde{f}_3^{3\omega} = \frac{(e\tau/\hbar)^3}{4(1 + i\omega\tau)(1 + 2i\omega\tau)(1 + 3i\omega\tau)} \mathcal{E}_\alpha \mathcal{E}_\beta \mathcal{E}_\gamma \partial_\alpha \partial_\beta \partial_\gamma f_0$$
$$\tilde{f}_3^\omega = \frac{3(e\tau/\hbar)^3}{4(1 - i\omega\tau)(1 + i\omega\tau)(1 + 2i\omega\tau)} \mathcal{E}_\alpha^* \mathcal{E}_\beta \mathcal{E}_\gamma \partial_\alpha \partial_\beta \partial_\gamma f_0 \tag{S5}$$

Having found the non-equilibrium distribution up to third order in the electric field intensity, we proceed to extracting the current densities. The total current density is given by

$$\begin{aligned} j_\alpha(t) &= \int_k -e v_\alpha(\mathbf{k}, t) f(\mathbf{k}, t) \\ &= -\frac{e}{\hbar} \int_k \partial_\alpha \varepsilon_\mathbf{k} f(\mathbf{k}, t) + \frac{e^2}{\hbar} \int_k \epsilon_{\alpha\beta\gamma} \Omega_\beta(\mathbf{k}) E_\gamma(t) f(\mathbf{k}, t) \end{aligned}$$

where $\int_k \equiv \int \frac{d^d k}{(2\pi)^d}$. We will thus obtain two types of terms at each order, a Drude-like term involving $\partial_\alpha \varepsilon$ and a moment of the Berry curvature Ω_β. At first order we have $j_\alpha^{(1)} = \mathrm{Re}\{\tilde{j}_\alpha^{(1)}\}$

$$\tilde{j}_\alpha^{(1)}(t) = \left(-\frac{e}{\hbar} \int_k \partial_\alpha \varepsilon_\mathbf{k} f_1^\omega + \frac{e^2}{\hbar} \int_k \epsilon_{\alpha\beta\gamma} \Omega_\beta \mathcal{E}_\gamma f_0 \right) e^{i\omega t} \tag{S6}$$

so we get a first-harmonic response. Note that for systems with TRS, the second term, representing the anomalous linear Hall conductivity, vanishes because the integrand is antisymmetric in $\mathbf{k}$. At second order, we instead have

$$\tilde{j}_\alpha^{(2)}(t) = -\frac{e}{\hbar} \int_k \partial_\alpha \varepsilon_\mathbf{k} (f_2^0 + f_2^{2\omega} e^{2i\omega t}) + \frac{e^2}{\hbar} \int_k \epsilon_{\alpha\beta\gamma} \Omega_\beta \mathcal{E}_\gamma f_1^\omega e^{2i\omega t} \tag{S7}$$

For systems with TRS, the Drude-like terms vanish because they involve an odd number of k-derivatives. Thus we expect the second-harmonic term to arise purely from the Berry dipole term. Finally, at third order we find that

$$\begin{aligned}\tilde{j}_\alpha^{(3)}(t) =& -\frac{e}{\hbar}\int_k \partial_\alpha \varepsilon_{\mathbf{k}} (f_3^{|\omega|} e^{i\omega t} + f_2^{3\omega} e^{3i\omega t}) \\ &+ \frac{e^2}{2\hbar}\int_k \epsilon_{\alpha\beta\gamma}\Omega_\beta \big[\big(\mathcal{E}_\gamma (f_2^0 + f_2^{0*}) + \mathcal{E}_\gamma^* f_2^{2\omega}\big) e^{i\omega t} \\ &\qquad + \mathcal{E}_\gamma f_2^{2\omega} e^{3i\omega t}\big] \end{aligned} \tag{S8}$$

so we get first and third harmonic response. Once again in systems with TRS, the terms in the second line vanish. To have a more complete treatment of non-linear response in the semiclassical formalism, we also include corrections to the single particle dispersion $\varepsilon_{\mathbf{k}}$ and the Berry curvature $\mathbf{\Omega}(\mathbf{k})$ due to the applied electric field. Following [3, 4], these corrections are:

$$\tilde{\varepsilon}_{\mathbf{k}} = \varepsilon_{\mathbf{k}} + e r_\alpha E_\alpha + \frac{e}{2} E_\alpha G_{\alpha\beta} E_\beta \tag{S9}$$

$$\tilde{\Omega}_\alpha = \Omega_\alpha + \epsilon_{\alpha\beta\gamma}\partial_\beta G_{\gamma\sigma} E_\sigma \tag{S10}$$

where we defined the Berry connection polarizability tensor (BCP) for a band (labelled "n") [5, 6] as:

$$G^n_{\alpha\beta} = 2e\,\mathrm{Re}\left\{ \sum_{m\neq 0} \frac{\mathcal{A}_\alpha^{0m}\mathcal{A}_\beta^{m0}}{\varepsilon_n - \varepsilon_m} \right\} \tag{S11}$$

where $\mathcal{A}_\alpha^{nm} = i\langle u_n|\partial_\alpha|u_m\rangle$ is the inter-band Berry connection. We note that the BCPT is related to the quantum metric $g^n_{\alpha\beta}$ via $G^n_{\alpha\beta} = -e\frac{\partial g^n_{\alpha\beta}}{\partial \epsilon_n}$. Consequently, we get two additional 3rd order contributions relevant for time-reversal invariant systems:

$$\begin{aligned}\mathrm{Re}\{\tilde{j}_\alpha^{(3),\mathrm{BCP}}(t)\} =& -\frac{e^2}{2\hbar}\int_k \partial_\alpha G_{\beta\gamma} E_\beta E_\gamma\,\mathrm{Re}\{\tilde{f}_1^\omega e^{i\omega t}\} \\ &+ \frac{e^2}{\hbar}\int_k \epsilon_{\alpha\beta\gamma}\epsilon_{\sigma\mu\beta}\partial_\sigma G_{\mu\delta} E_\delta E_\gamma\,\mathrm{Re}\{\tilde{f}_1^\omega e^{i\omega t}\}\end{aligned}$$

Note we don't need the third order correction to ϵ, because that would go into $\int_k \partial_\alpha \epsilon^{(3)} f_0$ which vanishes by TRS. Likewise we don't need the second order correction to Ω, because the anomalous Hall response vanishes. Therefore the treatment is fully consistent to third order in the electric field.

After some simplifications one finds for the modified BCP-induced current:

$$\begin{aligned}\tilde{j}_\alpha^{(3),\mathrm{BCP}}(t) =& -\frac{e^2}{2\hbar}\int_k (3\partial_\alpha G_{\beta\gamma} - 2\partial_\beta G_{\alpha\gamma})\big[e^{3i\omega t}\mathcal{E}_\beta\mathcal{E}_\gamma f_1^\omega \\ &+ e^{i\omega t}(\mathcal{E}_\beta^*\mathcal{E}_\gamma f_1^\omega + \mathcal{E}_\beta\mathcal{E}_\gamma^* f_1^\omega + \mathcal{E}_\beta\mathcal{E}_\gamma f_1^{\omega*})\big]\end{aligned} \tag{S12}$$

in agreement with the DC expression in [2].

To summarize, the current in a time-reversal symmetric electronic system subject to an external AC field $\mathbf{E}(t) = \mathrm{Re}\{\mathcal{E}e^{i\omega t}\}$ can be decomposed into powers of $\mathcal{E}$ as:

$$\mathbf{j}(t) = \mathrm{Re}\{\tilde{\mathbf{j}}_1 + \tilde{\mathbf{j}}_2 + \tilde{\mathbf{j}}_3 + ...\}$$

where

$$\tilde{j}_\alpha^{(1)}(t) = -\frac{e}{\hbar}\int_k \partial_\alpha \varepsilon_{\mathbf{k}} f_1^{\omega} e^{i\omega t} \tag{S13}$$

$$\tilde{j}_\alpha^{(2)}(t) = \frac{e^2}{\hbar}\int_k \epsilon_{\alpha\beta\gamma}\Omega_\beta \mathcal{E}_\gamma f_1^{\omega} e^{2i\omega t} \tag{S14}$$

$$\tilde{j}_\alpha^{(3)}(t) = -\frac{e}{\hbar}\int_k \partial_\alpha \varepsilon_{\mathbf{k}} (f_3^{\omega} e^{i\omega t} + f_3^{3\omega} e^{3i\omega t}) \tag{S15}$$

$$\begin{aligned}\tilde{j}_\alpha^{(3),\mathrm{BCP}}(t) = &-\frac{e^2}{2\hbar}\int_k (3\partial_\alpha G_{\beta\gamma} - 2\partial_\beta G_{\alpha\gamma})\big[e^{3i\omega t}\mathcal{E}_\beta\mathcal{E}_\gamma f_1^{\omega} \\ &+ e^{i\omega t}(\mathcal{E}_\beta^*\mathcal{E}_\gamma f_1^{\omega} + \mathcal{E}_\beta\mathcal{E}_\gamma^* f_1^{\omega} + \mathcal{E}_\beta\mathcal{E}_\gamma f_1^{\omega *})\big]\end{aligned} \tag{S16}$$

The definitions of the various $f_n^{m\omega}$ can be found in Eqns. (S3),(S4),(S5). These take into account the corrections to the energy dispersion and Berry curvature due to the presence of the electric field consistently up to third order.

2 Symmetry analysis for competing mechanisms

PdGa is in space group $P2_13$, which has 2-fold screw axes along the crystal axes, and 3-fold rotation axes through the body center. A set of generators are for example:

$$\{2_{001}|\frac{1}{2}\ 0\ \frac{1}{2}\},\ \{2_{010}|0\ \frac{1}{2}\ \frac{1}{2}\},\ \{3^+_{111}|0\} \tag{S17}$$

Let's consider the tensor $D_{\alpha\beta} = \int_{\mathbf{k}} \Omega_\alpha(\mathbf{k})\partial_\beta\epsilon_{\mathbf{k}}$, which is the product of an axial/pseudo-vector (the Berry curvature) with a cartesian vector. It therefore transforms itself as an axial tensor:

$$D \rightarrow D' = (\det R)\cdot RDR^T \tag{S18}$$

For space group $P2_13$, all crystal symmetries preserve handedness so $\det R = 1$. Then using the 2_{001} point-group symmetry, which transforms $(x, y, z) \rightarrow (-x, -y, z)$, we find that $D_{zx} = D_{zy} = 0$. Similarly using the other two two-fold axes we get that the off-diagonals of D must vanish. Finally, using of the 3-fold point-group symmetries, like 3^+_{111}, we find that $(x, y, z) \rightarrow (z, x, y)$ so that $D_{xx} = D_{yy} = D_{zz}$. Hence $D_{\alpha\beta} = D_0\delta_{\alpha\beta}$. It follows that $\tilde{j}_\alpha^{(2)}(t) \propto \epsilon_{\alpha\beta\gamma}D_0\delta_{\beta\sigma}E_\gamma E_\sigma = 0$, so the NLHE induced by the BCD vanishes by symmetry. Similar arguments can also be applied for other pseudo-vectors originated due to equilibrium bandstructure associated metric.

An applied electric field can break the material's inherent symmetries, but the consequences depend on its orientation. When the field is not aligned with high-symmetry directions like the screw axes or the body diagonal, it breaks both the 2_{001} point-group and the 3-fold rotational symmetries. Under these non-equilibrium conditions, the tensor in Eq. (S16) is permitted to have non-zero components.

Conversely, if the electric field is applied along a principal axis such as [001], the 3-fold rotational symmetry is still broken, but the 2_{001} point-group symmetry remains intact. The preservation of this symmetry prevents the formation of a net dipole from the Fermi pockets at Γ and R. As a result, there is no preferential scattering of fermions into any arm of the device, causing the NLH currents from each band to cancel out by symmetry.

This analysis aligns with experimental data below a threshold current, where the nonlinear response is observed to be zero (see Extended Data Fig. 3). Above this threshold, however,

a finite non-linear response appears. This onset occurs because the 2_{001} point-group symmetry is ultimately broken by a non-zero, band-normalized quantum metric, which emerges from the mesoscopic orbital magnetization of phase-coherent chiral currents. Modeling this non-equilibrium transition reveals a key limitation of semiclassical analysis, underscoring the necessity of including the metric associated with non-equilibrium bands.

References

[1] Inti Sodemann and Liang Fu. "Quantum nonlinear Hall effect induced by Berry curvature dipole in time-reversal invariant materials". In: *Physical review letters* 115.21 (2015), p. 216806.

[2] Tianyu Liu et al. "Quantum geometry in condensed matter". In: *National Science Review* 12.3 (2025), nwae334.

[3] Yang Gao, Shengyuan A Yang, and Qian Niu. "Field induced positional shift of Bloch electrons and its dynamical implications". In: *Physical review letters* 112.16 (2014), p. 166601.

[4] Huiying Liu et al. "Berry connection polarizability tensor and third-order Hall effect". In: *Physical Review B* 105.4 (2022), p. 045118.

[5] Chong Wang, Yang Gao, and Di Xiao. "Intrinsic nonlinear Hall effect in antiferromagnetic tetragonal CuMnAs". In: *Physical Review Letters* 127.27 (2021), p. 277201.

[6] Huiying Liu et al. "Intrinsic second-order anomalous Hall effect and its application in compensated antiferromagnets". In: *Physical Review Letters* 127.27 (2021), p. 277202.
